# Personalized TMS: role of RNA genotyping

**DOI:** 10.1108/MIJ-10-2019-0004

**Published:** 2019-11-04

**Authors:** Shawna Chan, Robert Bota

**Affiliations:** University of California Irvine, Irvine, California, USA

**Keywords:** Electroconvulsive therapy, Intermittent theta burst stimulation, Noninvasive brain stimulation, Transcranial direct current stimulation, Transcranial magnetic stimulation, Treatment-resistant psychiatric disorders

## Abstract

**Purpose:**

Noninvasive brain stimulation (NIBS) such a transcranial magnetic stimulation, intermittent theta burst stimulation, transcranial direct current stimulation and electroconvulsive therapy have emerged as an efficacious and well-tolerated therapy for treatment-resistant psychiatric disorders. While novel NIBS techniques are an exciting addition to the current repertoire of neuropsychiatric therapies, their success is somewhat limited by the wide range of treatment responses seen among treated patients.

**Design/methodology/approach:**

In this study, the authors will review the studies on relevant genetic polymorphisms and discuss the role of RNA genotyping in personalizing NIBS.

**Findings:**

Genome studies have revealed several genetic polymorphisms that may contribute for the heterogeneity of treatment response to NIBS where the presence of certain single nucleotide polymorphisms (SNPs) are associated with responders versus nonresponders.

**Originality/value:**

Historically, mental illnesses have been arguably some of the most challenging disorders to study and to treat because of the degree of biological variability across affected individuals, the role of genetic and epigenetic modifications, the diversity of clinical symptomatology and presentations and the interplay with environmental factors. In lieu of these challenges, there has been a push for personalized medicine in psychiatry that aims to optimize treatment response based on one’s unique characteristics.

## 1. Introduction to noninvasive brain stimulation

Noninvasive brain stimulation (NIBS) such a transcranial magnetic stimulation (TMS), intermittent theta burst stimulation (iTBS), transcranial direct current stimulation (tDCS) and electroconvulsive therapy (ECT) have emerged as an efficacious and well-tolerated therapy for treatment-resistant psychiatric disorders. These revolutionary neuromodulation techniques permit healthcare providers to alter cortical excitability without physically penetrating into brain tissue, such that sessions can be delivered in minutes on an outpatient basis, and patients may return to their daily activities on the same day. Additionally, these brain stimulation methods are postulated to induce changes on local neural activity that outlast the duration of stimulation and may be synergistically combined with pharmacotherapies to deliver therapeutic effects more quickly and with greater efficacy and durability than either modality alone (Rumi *et al.*, 2005; Rossini *et al.*, 2005; Liu *et al.*, 2014; Chen *et al.*, 2013; Bretlau *et al.*, 2008).

### 1.1 Transcranial magnetic stimulation (TMS)

TMS therapy utilizes a computerized medical device to generate magnetic resonance imaging-strength (MRI-strength) magnetic fields that pass through the skull and induce focal electric currents that depolarize axons in target areas of brain tissue, modulating local neural activity with downstream effects in neural networks throughout the cortex (Lefaucheur *et al.*, 2014; Allan *et al.*, 2012). Daily TMS treatments are delivered over the course of 4-6 weeks, and as they do not require anesthetic agents (as in ECT), patients are able to drive themselves to and from daily appointments. TMS was first recognized for its role in acute treatment of major depressive disorder (MDD) resistant to treatment with antidepressant medication or in patients unable to tolerate antidepressant medication. For the treatment of MDD, the strongest evidence exists for application of high-frequency TMS over the left dorsal lateral prefrontal cortex (DLPFC), and notably left DLPFC hypoactivity has been found on neuroimaging of patients with MDD (Baeken and De Raedt, 2011). The efficacy and safety of TMS have been demonstrated in various large, multisite randomized clinical trials, and it has been shown to produce clinically meaningful improvement in depressive symptoms as well significant improvement in quality of life; benefits were observed immediately following treatment with TMS and at follow-up 6-12 months later (O’Reardon *et al.*, 2007; George *et al.*, 2010; Levkovitz *et al.*, 2015; Solvason *et al.*, 2014). Safety considerations in the use of TMS include the development of headache or scalp discomfort at the site of application, hearing loss that may be prevented with hearing protection, a low incidence of seizures (lower than the risk reported for use of antidepressant medications) and vasovagal syncope (Janicak *et al.*, 2008; Rossi *et al.*, 2009; Tringali *et al.*, 2012; Anderson *et al.*, 2009).

Currently, TMS is approved by the Food and Drug Administration (FDA) for treatment of treatment-resistant major depression (O’Reardon *et al.*, 2007; George *et al.*, 2010; Levkovitz *et al.*, 2015), pain associated with migraine headaches (Starling *et al.*, 2018; Lipton *et al.*, 2010; Bhola *et al.*, 2015) and treatment-resistant obsessive-compulsive disorder (OCD) (Hawken *et al.*, 2016). Investigations of its therapeutic role in various other neuropsychiatric disorders, including schizophrenia, bipolar disorder, Parkinson’s disease, stroke-related deficits and chronic pain, are currently underway (Lefaucheur *et al.*, 2014; He *et al.*, 2017; Quan *et al.*, 2015; Wobrock *et al.*, 2015; Tavares *et al.*, 2017; Hu *et al.*, 2016; Cohen *et al.*, 2018; Brys *et al.*, 2016; Zheng *et al.*, 2015; Ludemann-Podubecka *et al.*, 2015; Attal *et al.*, 2016; Sankarasubramanian *et al.*, 2017).

### 1.2 Intermittent theta burst stimulation (iTBS)

iTBS is a more recent form of TMS that delivers 50 Hz bursts applied at 5 Hz that mimic endogenous hippocampal theta rhythms (Larson and Munkacsy, 2015). This approach has the advantage of delivering maximal synaptic long-term potentiation to hippocampal neurons, thereby enhancing the efficiency of neuromodulation by exerting longer-lasting effects on motor cortex excitability while requiring shorter treatment sessions (3 versus 38 min) than conventional rTMS (Blumberger *et al.*, 2018). The outcomes of iTBS have been shown to be equal to TMS for the treatment of MDD with a similar safety and tolerability profile where increased headache pain scores were reported with iTBS (Blumberger *et al.*, 2018). Currently, iTBS is approved by the FDA for treatment-resistant MDD.

### 1.3 Transcranial direct current stimulation (tDCS)

tDCS is an investigational neuromodulation technique only available through research protocols that delivers a low-intensity current to specific cortical regions, typically the left DLPFC (Boggio *et al.*, 2008). Instead of causing neuronal depolarization as in TMS, tDCS modulates spontaneous neuronal firing to modulate cortical excitatory tone (Nitsche *et al.*, 2008). Anodal stimulation is associated with increased cortical excitability, while cathodal stimulation is associated with decreased cortical excitability, with effects potentially lasting beyond the period of stimulation (Tortella *et al.*, 2015). For the treatment of depression, tDCS is theorized to modulate neural networks of cortical regions involved in mood regulation. Although some studies report that tDCS may be as effective as rTMS and antidepressant pharmacotherapy (Brunoni *et al.*, 2016; Kalu *et al.*, 2012; Berlim *et al.*, 2013), notable side effects include the development of skin burns and the risk of switching from depression to hypomania (Loo *et al.*, 2011; Loo *et al.*, 2012; Brunoni *et al.*, 2017).

### 1.4 Personalized medicine: genomic data allow us to predict response for rapid treatment optimization for patients with refractory illnesses

While novel NIBS techniques are an exciting addition to the current repertoire of neuropsychiatric therapies, their success is somewhat limited by the wide range of treatment responses seen among treated patients. Historically, mental illnesses have been arguably some of the most challenging disorders to study and to treat because of the degree of biological variability across affected individuals, the role of genetic and epigenetic modifications, the diversity of clinical symptomatology and presentations and the interplay with environmental factors. In lieu of these challenges, there has been a push for personalized medicine in psychiatry that aims to optimize treatment response based on one’s unique characteristics. Genome studies have revealed several genetic polymorphisms that may contribute for the heterogeneity of treatment response to NIBS where the presence of certain SNPs are associated with responders versus non-responders. In this article, we will review the studies on relevant genetic polymorphisms and discuss the role of RNA genotyping in personalizing NIBS.

## 2. Search strategy

We searched PubMed, the primary biomedical database, between 2000 and October 2018, for literature relevant to topic of this review article. The search terms that were used included “transcranial magnetic stimulation,” “transcranial direct stimulation,” “theta burst stimulation,” “polymorphism,” “genomic” and “genotype.” Bibliographies of articles were further hand-searched to identify additional relevant articles. Only published, peer-reviewed articles available in English were considered for this review.

## 3. Significant genes and their polymorphisms

### 3.1 Brain-derived neurotrophic factor (Val66Met)

Brain-derived neurotrophic factor (BDNF) gene encodes BDNF protein, which plays an important role in neuronal growth and differentiation, synaptic transmission, neuroprotection and neuroplasticity. BNDF binds tropomyosin receptor kinase B (TrkB) and activates signaling cascades, including the Ras/MAPK-ERK, IRS-1/P13K/AKT and PLC/DAG/IP3 pathways, which activate downstream survival and growth genes (Bathina and Das, 2015). BDNF protein is highly expressed throughout various brain structures, including the hippocampus, basal forebrain, cortex and hypothalamus, and is implicated in learning, memory and higher cognitive function (Lu *et al.*, 2014; Cunha *et al.*, 2010). Deficits in BDNF signaling have been associated with predisposition toward neuropsychiatric disorders such as depression, bipolar disorder, Alzheimer’s disease, Parkinson’s disease and Huntington’s disease (Binder and Scharfman, 2004). The BDNF Val66Met SNP is caused by a G > A point mutation at position 196, which destabilizes BDNF mRNA and interferes with trafficking and protein release (Wu *et al.*, 2011). This polymorphism has been linked to hippocampal and cortical atrophy with the disruption of neural networks that traverse these areas. In the context of neuromodulation techniques that affect neural activity and excitatory tone at a local and network level, it is reasonable that the Val66Met SNP would influence treatment response.

BDNF is believed to be an important regulator rehabilitation-induced recovery following stroke by enhancing synaptic plasticity, increasing angiogenesis and neurogenesis and stimulating brain repair (Di Lazzaro *et al.*, 2015; Berretta *et al.*, 2014; Mirowska-Guzel *et al.*, 2013). In stroke patients treated with TMS, the Val66Met polymorphism has been associated with poorer recovery of motor function compared to the homozygous ValVal genotype (Chang *et al.*, 2014). The mechanism of how the Val66Met polymorphism interacts with NIBS in stroke rehabilitation remains to be found as inconsistent results are reported. In a study of 20 patients who had suffered from first-ever ischemic stroke, ValVal patients were found to have greater excitability over the unaffected hemisphere and increased inter-hemispheric imbalance compared to ValMet patients, and the difference became even more pronounced after application of iTBS; no difference in motor-evoked potential (MEP) threshold nor amplitude were noted between the two genotypes (Di Lazzaro *et al.*, 2015). A study of 22 chronic stroke patients found that ValVal patients showed a significantly higher increase of MEP amplitude following treatment with high-frequency TMS over the ipsilesional M1 (Uhm *et al.*, 2015); however, when evaluated in healthy volunteers and schizophrenic patients, the Val66Met polymorphism was not found to differentially influence MEP following TMS and tDCS, respectively (Hwang *et al.*, 2015; Strube *et al.*, 2014).

In patients suffering from major depression, Val66Met polymorphism appears to negatively impact treatment response to TMS. In a study of 36 patients with drug-resistant depression, TMS treatment significantly improved depression symptoms as assessed by the Hamilton rating scale for depression (HAMD) in ValVal patients versus ValMet patients (Bocchio-Chiavetto *et al.*, 2008). Additionally, in a study of 19 female patients with pharmacoresistant MDD treated with TMS, 80 per cent of patients who sustained significant HAMD score reduction at six months were of the ValVal genotype (Krstic *et al.*, 2014). ValVal patients appear to show consistently better responses to TMS for MDD, and the mechanism may have to do with differences in modulation of motor cortex excitability and susceptibilities of synapses to undergo long-term potentiation (LTP) and long-term depression (LTD) (Cheeran *et al.*, 2008; Cirillo *et al.*, 2012). Interestingly, in a study of 40 healthy volunteers, the effect of BDNF polymorphism on cortical excitability following high-frequency TMS was only observed after delivery of sub-threshold intensity (90 per cent of resting motor threshold (rMT)), but not supra-threshold intensity (110 per cent of rMT); no significant side effects were observed between the treatments. This finding suggests the mechanism may be even more complex, where BDNF polymorphism affects the threshold for likelihood of TMS-induced changes, with differences more likely to be observed when delivering weaker intensity. If this were the case, the treatment plan for patients with known Val66Met polymorphism may be safely adjusted to increase likelihood of treatment response to TMS. However, Val66Met polymorphism was not found to impact treatment response to tDCS (Brunoni *et al.*, 2013).

### 3.2 Dopamine D2 receptor (–957 C > T) and catechol-O-metyltransferase (Val158Met)

The dopamine D2 receptor (DRD2) gene encodes a G-protein-coupled receptor that is highly expressed in the striatum and nucleus accumbens that mediates a variety of cognitive functions, including learning and memory, attention, reward behavior and pain response (Gluskin and Mickey, 2016). The most frequently studied DRD2 genetic variant is the Glu713Lys polymorphism caused by a missense C > T mutation which appears to affect D2 receptor binding potential (Wagner *et al.*, 2014; Savitz *et al.*, 2013), receptor availability (Smith *et al.*, 2017; Hirvonen *et al.*, 2009) and mRNA stability and synthesis of the D2 receptor (Duan *et al.*, 2003). Aberrant dopamine signaling is involved in the pathogenesis of various mental illnesses, including schizophrenia, bipolar disorder, Parkinson’s disease, Huntington’s disease and attention deficit disorder (Beaulieu and Gainetdinov, 2011), and dopamine receptors are frequently pharmacologic targets in the treatment of psychiatric conditions.

Because treatment with NIBS involves long-term modulation of cortical excitability, DRD2 variants are especially relevant in patients receiving NIBS because the D2 receptor has been shown to have dose-dependent effects on neuroplasticity. In healthy adults, D2 receptor blockade with sulpiride abolished tDCS-induced changes of excitability in both anodal and cathodal tDCS after-effects 5 min following stimulation (Nitsche *et al.*, 2006). A similar effect was seen in healthy adult undergoing TBS, where administration of sulpiride blocked the excitatory effects of iTBS and inhibitory effects of continuous TBS (Monte-Silva *et al.*, 2011), further supporting the important role of DRD2 in synaptic plasticity and NIBS outcomes.

For the treatment of neuropathic pain, rTMS of the motor cortex has been shown to produce analgesic effects via activation of the endogenous opioid network (de Andrade *et al.*, 2011; Maarrawi *et al.*, 2007). Meanwhile, DRD2 availability is known to be involved in pain modulatory capacity and response to pain (Pertovaara *et al.*, 2004; Hagelberg *et al.*, 2002); hence, activation of the striatal dopamine system may lead to release of endogenous opioids. In a study of healthy subjects and patients with neuropathic orofacial pain genotyped for the DRD2 gene 957 C>T polymorphism, healthy subjects homozygous for the DRD2 957 T allele had increased thermal baseline sensitivity of facial skin measured by thermal detection threshold; additionally, rTMS to the sensorimotor cortex was only able to produce an analgesic effect in healthy 957TT homozygotes. Meanwhile, the prevalence of the 957TT allele was higher in patients with chronic neuropathic orofacial pain when compared with the general population, and patients with the 957TT allele experienced more severe pain symptoms. The authors suggest that DRD2 polymorphisms are not only implicated in pain sensitivity and susceptibility to chronic neuropathic pain but also in the analgesic efficacy of rTMS (Jaaskelainen *et al.*, 2014). However, the clinical significance of DRD2 957 C>T polymorphism remains unclear, as upon evaluation of clinical symptoms through Brief Pain Inventory scores, no correlation was found between genetic polymorphisms and patient-reported analgesia following treatment with rTMS in the same subset of chronic pain patients (Lindholm *et al.*, 2015).

The catechol-O-methyltransferase (COMT) enzyme has been hypothesized to play a role in DRD2 variants because of its function in metabolizing and degrading dopamine. The COMT missense variant Val158Met modulates COMT activity and subsequently decreased dopamine levels in the DLPFC, where the Val/Val carriers demonstrate the most rapid dopamine metabolism, Val/Met carriers demonstrate intermediate dopamine metabolism and Met/Met carriers demonstrate the slowest metabolism (Chen *et al.*, 2004). COMT activity is clinically relevant as higher enzymatic activity may lead to lower DLPFC dopamine signaling and impairment of cortical function. In a study of healthy adults, COMT Met/Met carriers were found to have lower set-shifting ability after anodal tDCS to DLPFC (Plewnia *et al.*, 2013). Response inhibition, an important component of executive function, was found to be significantly impaired in healthy COMT Val/Val carriers after cathodal tDCS to DLPFC, while no effect of cathodal stimulation was seen in healthy Met-carriers (Nieratschker *et al.*, 2015). The COMT genotype has also been found to interact with tDCS-linked working memory performance and tDCS intensity, where Val/Val homozygotes showed improved visual working memory and spatial working memory after receiving intermediate intensity anodal frontoparietal tDCS and low-intensity tDCS, respectively, while Met/Met homozygotes showed decreased spatial working memory performance following high-intensity tDCS (Stephens *et al.*, 2017).

Interestingly, in patients with schizophrenia, homozygosity for COMT Val/Val is associated with significantly greater reduction of auditory hallucinations following tDCS to DLPFC compared to Val/Met and Met/Met carriers (Shivakumar *et al.*, 2015; Chhabra *et al.*, 2018). In the same cohort of patients, the authors also found that the neuregulin-1 (NRG1) rs35753505 polymorphism, which has been studied extensively for its potential role in the pathogenesis of schizophrenia, further decreased auditory hallucinations when present with COMT Val/Val. These findings suggest that the COMT Val158Met polymorphism influences the effect of tDCS on cognitive flexibility, which may ultimately lead to different levels of clinic benefit dependent on one’s COMT genotype. Together, the DRD2 G > T and COMT Val158Met polymorphisms have been shown to affect important cognitive functions such as motor learning and performance, working memory and executive function (Klaus *et al.*, 2017; Xu *et al.*, 2007a; Noohi *et al.*, 2014); their genetic effects may impact clinical outcomes in patients receiving NIBS and should be considered when determining optimal dosing and treatment regimens.

### 3.3 5-HT1A (rs6295) and 5-HHT (SERTPR/5-HTTLPR)

The serotonergic system regulates a diverse range of cognitive functions, including mood, sexual behavior, learning and memory. The role of serotonin, also known as 5-hydroxytryptamine (5-HT), has been widely studied in the pathogenesis of major depressive disorder in which the monoamine hypothesis of depression states that serotonin deficiency is linked with depression. Repetitive TMS to the DLPFC is currently an adjuvant treatment for treatment-resistant depression and theoretically enhances neuronal activity of serotonergic and/or dopaminergic systems. Animal studies have shown increased levels of 5-HT in the hippocampus and amygdala (Juckel *et al.*, 1999), 5-HT receptor modulation throughout the brain (Gur *et al.*, 2000; Ben-Shachar *et al.*, 1999) and changes in 5-HT metabolism (Sibon *et al.*, 2007) in limbic areas following TMS. Genetic polymorphisms that may affect antidepressant responses to TMS include: the short (s) and long (l) variants of the serotonin transporter promoter region (SERTPR/5-HTTLPR), where the long variant has longer basal activity and may facilitate better response to selective serotonin reuptake inhibitors (SSRIs) (Serretti *et al.*, 2007; Porcelli *et al.*, 2012; Zanardi *et al.*, 2001; Durham *et al.*, 2004), and a G to C substitution at –1019 of the 5-HT1A serotonergic receptor promoter region (rs6295), where C/C homozygotes demonstrated better response to SSRIs, while G/G homozygotes were over-represented in depressed patients when compared to controls (Hong *et al.*, 2006; Arias *et al.*, 2005; Lemonde *et al.*, 2003).

Several studies have evaluated these polymorphisms in the context of NIBS for the treatment of depression. In a study in patients with a history of major depressive disorder, patients with the l allele of 5-HTT showed greater Hamilton Depression Rating Scale (HDRS) score reduction than s/s homozygotes, although the influence of the 5-HTT genotype was the same between active and sham TMS (Zanardi *et al.*, 2007). The authors suggested that perhaps the 5-HTT genotype not only affects treatment response but also duration of symptoms; therefore, improvement of symptoms could also be observed in placebo treatment (Bocchio-Chiavetto *et al.*, 2008). A separate study of patients with treatment-resistance depression also found greater HDRS score reduction in l/l homozygotes following rTMS; however in this study, no influence of the 5-HTT polymorphism was seen following sham treatment. For the 5-HT1A polymorphism, studies disorder have shown that following rTMS to the left DLPFC, patients with the C/C genotype had significantly greater improvement of depression as evaluated by the HDRS for depression than G/G and C/G individuals (Zanardi *et al.*, 2007; Malaguti *et al.*, 2011). Further studies are needed to clarify the utility of the 5-HTT and 5-HT1A polymorphisms to optimize NIBS outcomes for patients with treatment-resistance depression, and existing studies suggest these polymorphisms may be potential genetic predictors of treatment efficacy.

### 3.4 TRPV1 (rs222747 and rs222749)

The transient receptor potential vanillioid (TRPV1) gene encodes for nonselective cation channels that regulate glutamate release in response to a variety of stimuli such as endocannabinoids, eicosanoids, vanilloid compounds, voltage and heat (Suh and Oh, 2005; De Petrocellis and Marzo, 2005). TRPV1 has been shown to be important in synaptic plasticity, anxiety and fear and hippocampal long-term potentiation and may also be involved long-term depression (Marsch *et al.*, 2007; Maione *et al.*, 2009; Gibson *et al.*, 2008). The “G” allele of rs222747 polymorphism leads to increased TRPV1 mRNA and protein expression on the cell surface as well as increased glutamate release in response, while rs222749 polymorphism does not appear to alter the functionality of the TRPV1 channel (Xu *et al.*, 2007b). Following TMS to the right primary motor cortex, healthy adults with the G/G variant of rs222747 demonstrated significantly greater short-interval intracortical facilitation, which is believed to mirror the activation of glutamatergic cortical interneurons, while allelic variants of rs222749 did not show differences in cortical response to TMS (Mori *et al.*, 2012). Hence, TRPV1 channels may also be implicated in cortical excitability with some polymorphisms facilitating greater cortical response to NIBS than others.

### 3.5 GRN

Granulin (GRN) encodes progranulin, a secreted neuroregulatory growth factor believed to play a central role in brain development and neurodegeneration (van Swieten and Heutink, 2008; Petkau *et al.*, 2012). Various mice and human studies have demonstrated that progranulin insufficiency is associated with familial frontotemporal dementia (FTD), where structural changes such as decreased brain volumes and dendritic density as well as reduced functional connectivity were observed prior to the onset of FTD symptoms (Borroni *et al.*, 2008; Pievani *et al.*, 2014; Dopper *et al.*, 2014; Rohrer *et al.*, 2015). In a study of pre-symptomatic GRN mutation carriers (g.1977_1980delCACT and IVS6 + 5_8delGTGA) and nonmutation carriers, TMS to the primary hand motor cortex demonstrated impaired short-interval intracortical inhibition (SICI) and intracortical facilitation (ICF) of the right insula among GRN mutation carriers. Interestingly, brain MRI also noted increased cortical thickness in the supramarginal and superior parietal gyri and decreased surface area in the precuneus and inferior partial gyrus for GRN mutation carriers, which inversely correlated with SICI/ICF of the right insula (Gazzina *et al.*, 2018) ◊ GABA/glutamatergic impairment.

### 3.6 GRIN1 (rs4880213) and GRIN2B (rs1805247), SNCA (Ala53Thr), SCNA1, DAT1, GAG (rs11789969)
